# Evaluation in Mice of a Conjugate Vaccine for Cholera Made from *Vibrio cholerae* O1 (Ogawa) O-Specific Polysaccharide

**DOI:** 10.1371/journal.pntd.0002683

**Published:** 2014-02-06

**Authors:** Mohammad Murshid Alam, Megan Kelly Bufano, Peng Xu, Anuj Kalsy, Y. Yu, Y. Wu Freeman, Tania Sultana, Md. Rasheduzzaman Rashu, Ishaan Desai, Grace Eckhoff, Daniel T. Leung, Richelle C. Charles, Regina C. LaRocque, Jason B. Harris, John D. Clements, Stephen B. Calderwood, Firdausi Qadri, W. F. Vann, Pavol Kováč, Edward T. Ryan

**Affiliations:** 1 Division of Infectious Diseases, Massachusetts General Hospital, Boston, Massachusetts, United States of America; 2 International Centre for Diarrheal Disease Research, Bangladesh (ICDDR,B), Dhaka, Bangladesh; 3 NIDDK, LBC, National Institutes of Health, Bethesda, Maryland, United States of America; 4 Department of Medicine, Harvard Medical School, Boston, Massachusetts, United States of America; 5 Department of Pediatrics, Harvard Medical School, Boston, Massachusetts, United States of America; 6 Tulane University School of Medicine, New Orleans, Louisiana, United States of America; 7 Department of Microbiology and Immunobiology, Harvard Medical School, Boston, Massachusetts, United States of America; 8 CBER, FDA, Laboratory of Bacterial Toxins, Bethesda, Maryland, United States of America; 9 Department of Immunology and Infectious Diseases, Harvard School of Public Health, Boston, Massachusetts, United States of America; University of California San Diego School of Medicine, United States of America

## Abstract

**Background:**

Protective immunity against cholera is serogroup specific. Serogroup specificity in *Vibrio cholerae* is determined by the O-specific polysaccharide (OSP) of lipopolysaccharide (LPS). Generally, polysaccharides are poorly immunogenic, especially in young children.

**Methodology:**

Here we report the evaluation in mice of a conjugate vaccine for cholera (OSP:TThc) made from *V. cholerae* O1 Ogawa O-Specific Polysaccharide–core (OSP) and recombinant tetanus toxoid heavy chain fragment (TThc). We immunized mice intramuscularly on days 0, 21, and 42 with OSP:TThc or OSP only, with or without dmLT, a non-toxigenic immunoadjuvant derived from heat labile toxin of *Escherichia coli*.

**Principal Findings:**

We detected significant serum IgG antibody responses targeting OSP following a single immunization in mice receiving OSP:TThc with or without adjuvant. Anti-LPS IgG responses were detected following a second immunization in these cohorts. No anti-OSP or anti-LPS IgG responses were detected at any time in animals receiving un-conjugated OSP with or without immunoadjuvant, and in animals receiving immunoadjuvant alone. Responses were highest following immunization with adjuvant. Serum anti-OSP IgM responses were detected in mice receiving OSP:TThc with or without immunoadjuvant, and in mice receiving unconjugated OSP. Serum anti-LPS IgM and vibriocidal responses were detected in all vaccine cohorts except in mice receiving immunoadjuvant alone. No significant IgA anti-OSP or anti-LPS responses developed in any group. Administration of OSP:TThc and adjuvant also induced memory B cell responses targeting OSP and resulted in 95% protective efficacy in a mouse lethality cholera challenge model.

**Conclusion:**

We describe a protectively immunogenic cholera conjugate in mice. Development of a cholera conjugate vaccine could assist in inducing long-term protective immunity, especially in young children who respond poorly to polysaccharide antigens.

## Author Summary

Cholera is a severe dehydrating diarrheal illness of humans caused by organisms *Vibrio cholerae* serogroups O1 or O139 serogroup organisms. Protective immunity against cholera is serogroup specific. Serogroup specificity in *V. cholerae* is determined by the O-specific polysaccharide (OSP) of lipopolysaccharide (LPS). Generally, polysaccharides are poorly immunogenic, especially in young children. Unfortunately, children bear a large burden of cholera globally. Here we describe a novel cholera conjugate vaccine and show that it induces immune responses in mice, including memory responses, to OSP, the T cell-independent antigen that probably is the target of protective immunity to cholera. These responses were highest following immunization of the vaccine with a novel immunoadjuvant, dmLT. We also show that immunization of mice with this conjugate vaccine protects against challenge with wild-type *V. cholerae*. A protectively immunogenic cholera conjugate vaccine that induces long-term memory responses could have particular utility in young children who are most at risk of cholera.

## Introduction

Cholera is a severe dehydrating diarrheal illness of humans caused by organisms *Vibrio cholerae* O1 or O139 serogroup organisms. *V. cholerae* O139 has largely disappeared and is reported from just a few Asian countries [1]. Cholera affects 3–5 million people each year, killing ∼100,000 annually, and cholera is endemic in over 50 countries [2]. *V. cholerae* O1 can be distinguished genotypically and phenotypically into classical and El Tor biotypes [2] and Ogawa and Inaba serotypes. Ogawa differs from Inaba only by the presence of a 2-O-methyl group in the non-reducing terminal sugar of O-specific polysaccharide (OSP) [3]–[5]. Currently, the global cholera pandemic is caused by organisms *V. cholerae* O1, El Tor, organisms, with the prevalent serotype fluctuating during cholera outbreaks, switching between Ogawa and Inaba [1].

Protection against cholera is serogroup specific. Previous infection with *V. cholerae* O139 provides no cross-protection from cholera caused by *V. cholerae* O1, and vice versa [6]–[8]. Serogroup specificity is largely determined by the O-specific polysaccharide (OSP) of lipopolysacharide (LPS). OSP is attached to lipid A that is part of the outer membrane of *V. cholerae* [9]. We have previously shown that a synthetic neoglyconjugate cholera vaccine containing a hexasaccharide of *V. cholerae* O1 Ogawa is protectively immunogenic in mice [10]–[12]. We were therefore interested in evaluating whether a cholera conjugate vaccine containing native OSP recovered from *V. cholerae* O1 would also be immunogenic.

## Materials and Methods

### Ethics statement

The use of animals complied fully with relevant governmental and institutional requirements, guidelines, and policies. This work was approved by the Massachusetts General Hospital Subcommittee on Research Animal Care (SRAC) – OLAW Assurance # A3596-01; Protocol #2004N000192. The work adheres to the USDA Animal Welfare Act, PHS Policy on Humane Care and Use of Laboratory Animals, and the “ILAR Guide for the Care and Use of Laboratory Animals”.

### Bacterial strains and media

*V. cholerae* O1 El Tor Ogawa strain X25049 [13] was used to prepare LPS for use in vaccine preparation and immunological assays, in addition to vibriocidal assays, and wild-type classical *V. cholerae* O1 classical Ogawa strain O395 [10] was used in vibriocidal assays and the neonatal challenge. Strains were grown in Luria-Bertani broth.

### Vaccine antigen

LPS was recovered from X25049, and OSP-core (OSPc) was derived from LPS as previously described [9], [14]. As a carrier protein, recombinant tetanus toxoid heavy chain fragment (TThc) was used [15], [16]. TThc was prepared as a 52,108 Da recombinant protein in *E. coli* BL21 (DE3) Star with a self-cleaving intein tag using affinity and size exclusion chromatography, as previously described [17].

Conjugation was carried out as previously described [14]. Briefly, 3,4-dimethoxy-3-cyclobutene-1, 2-dione (4.0 mg) was added to a solution of Ogawa O-SP–core antigen (8.0 mg) in pH 7 phosphate buffer (0.05 M, 400 µL) contained in a 2 mL V-shaped reaction vessel, and the mixture was gently stirred at room temperature for 48 h. The solution was transferred into an Amicon Ultra (4 mL, 3K cutoff) centrifuge tube and dialyzed against pure water (centrifugation at 4°C, 7,500× g, 8 times, 35 min each time). The retentate was lyophilized to afford the O-SP–core squarate monomethyl ester as white solid (7.4 mg, 91%).

**Figure 1:**
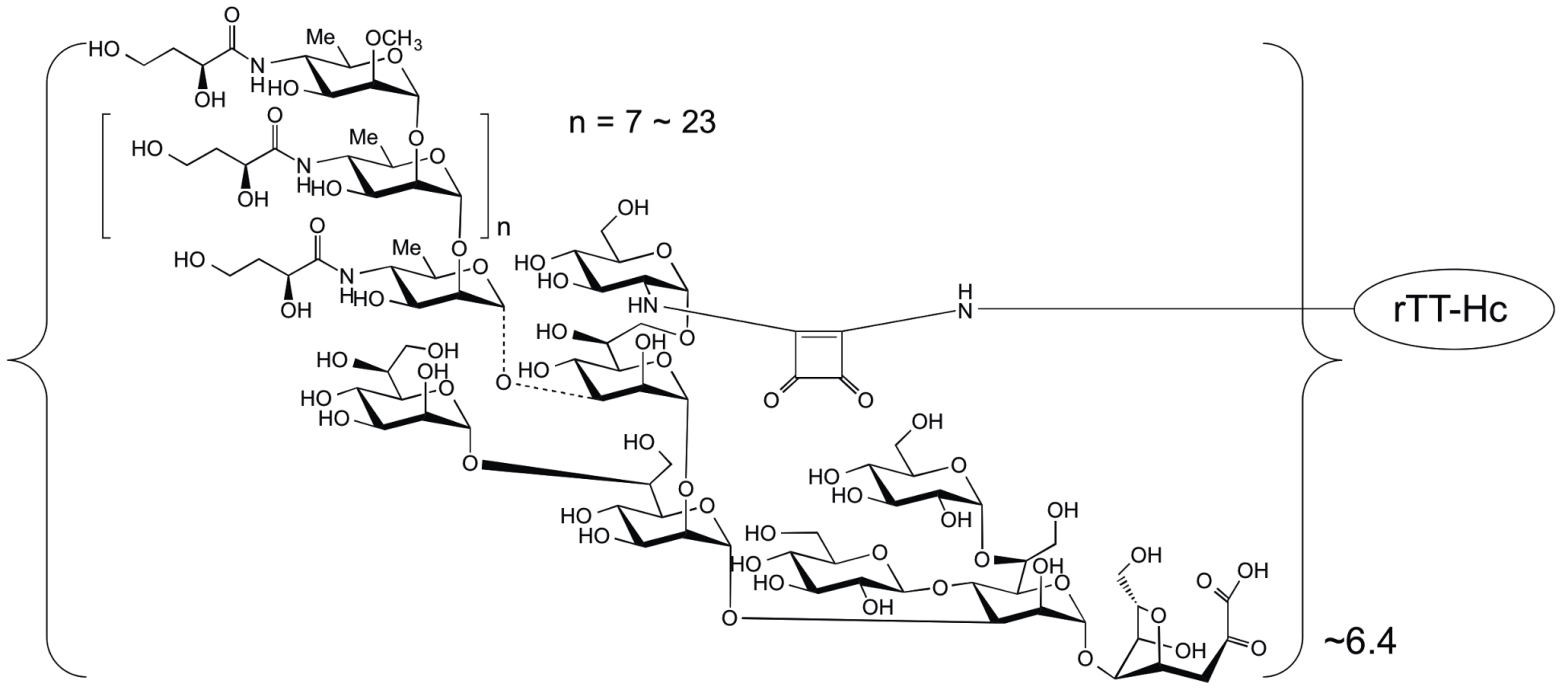
Structure of Ogawa OSP:TThc conjugate. 6.4 moles OSP per mole conjugate. TThc = recombinant tetanus toxoid heavy chain fragment.

TThc (3.2 mg) and the methyl squarate derivative of the Ogawa O-SP–core antigen described above (7.4 mg) were weighed into a 1 mL V-shaped reaction vessel and 240 µL of 0.5 M pH 9 borate buffer was added (to form ∼5 mM solution with respect to the antigen; antigen/carrier = 20∶1). A clear solution was formed. The mixture was stirred at room temperature and the progress of the reaction was monitored by SELDI-TOF MS at 24, 48, 72, 96, and 168 h, when no more increase of antigen/carrier ratio could be observed. The mixture was transferred into an Amicon Ultra (4 mL, 30 K cutoff) centrifuge tube and dialyzed (centrifugation at 4°C, 7,500× g, 8 times, 8 min each time) against 10 mM aqueous ammonium carbonate. After lyophilization, 4.6 mg (83%, based on TThc) of conjugate was obtained as a white solid. On the basis of the molecular mass of the carrier (52,108 Da), conjugate (90,000 Da, determined by SELDI TOF MS) and average MW of the OSP antigen of 5,900 Da [14], the antigen/TThc ratio was 6.4∶1 (conjugation efficiency, 32%) (figure 1). A corresponding conjugate was made of OSP: bovine serum albumin (BSA; Sigma #A-4503) using the same approach as described above for use in immunologic assays. The OSP:BSA product contained 4.8 moles OSP per BSA.

For these experiments, we used dmLT, a double mutant derivative of *Escherichia coli* heat labile toxin (LT), as an immunoadjuvant. dmLT (R192G/L211A) retains immunoadjuvanticity with markedly reduced enterotoxicity [18]. dmLT was prepared as previously described [18], [19].

### Immunization of mice and sampling

We immunized cohorts of 10–15, three to five week old Swiss Webster female mice intramuscularly with OSP:TThc or OSP (10 µg sugar per mouse; total 3 doses) with or without dmLT (5 µg). Mice were immunized on days 0, 21, and 42. We collected blood samples via tail bleeds on days 0, 21, 28, 42, 49 and 56. Samples were collected, processed, aliquoted, and stored as previously described [10], [11]. For the memory B cell assay, splenocytes were isolated after day 56 and processed for ELISPOT as previously described [20].

### Detection of specific antibody responses in serum

We quantified anti-LPS and OSP IgG, IgM and IgA responses in serum using standard enzyme-linked immunosorbent assay (ELISA) protocols [10], [11]. To assess anti-LPS antibody responses, we coated ELISA plates with *V. cholerae* O1 Ogawa LPS (2.5 µg/mL) in PBS [10], [11]. To assess anti-OSP antibody responses, we coated ELISA plates with OSP:BSA (1 µg/mL) in PBS. To each well, we added 100 µL of serum (diluted 1∶25 in 0.1% BSA in phosphate buffered saline-Tween) and detected the presence of antigen-specific antibodies using horseradish peroxidase-conjugated anti-mouse IgG, IgM or IgA antibody (diluted 1∶1000 in 0.1% BSA in phosphate buffered saline-Tween) (Southern Biotech, Birmingham, AL). After 1.5 h incubation at 37°C, we developed the plates with a 0.55 mg/mL solution of 2,2′ 0-azinobis (3-ethylbenzothiazoline-6-sulfonic acid) (ABTS; Sigma) with 0.03% H_2_O_2_ (Sigma), and determined the optical density at 405 nm with a Vmax microplate kinetic reader (Molecular Devices Corp. Sunnyvale, CA). Plates were read for 5 min at 30 s intervals, and the maximum slope for an optical density change of 0.2 U was reported as millioptical density units per minute (mOD/min). We normalized ELISA units (EU) by calculating the ratio of the optical density of the test sample to that of a standard of pooled sera from mice vaccinated with cholera vaccine from a previous study run on the same plate. We characterized a responder as a ≥2-fold increase in anti-LPS and OSP EU kinetic responses.

### Measurement of serum vibriocidal responses

We assessed serum vibriocidal antibody titers against *V. cholerae* X25049 in a micro-assay as previously described [21], [22]. We inactivated endogenous complement activity of mouse serum by heating it for 1 hr at 56°C. We then added 50 µl aliquots of two-fold serial dilutions of heat-inactivated sera in 0.15M saline (1∶25 to 1∶25,600) to wells of sterile 96-well tissue culture plates containing 50 µl/well of *V. cholerae* X25049 (OD 0.1) in 0.15M saline and 22% guinea pig complement (EMD Biosciences, San Diego, CA). The plates were then incubated for 1 hr at 37°C. 150 µl of brain heart infusion broth (Becton Dickinson, Sparks, MD) was added to each well, and plates were incubated for an additional 1.5 h at 37°C, when optical density at 600 nm was assessed. We calculated the vibriocidal titer as the dilution of serum causing 50% reduction in optical density compared with that of wells containing no serum [23], [24]. We characterized a responder as a ≥4-fold increase in vibriocidal titer.

### Memory B cell responses

We assessed memory B-cell assays after the third round of immunization based on previously described methods [20]. Briefly, we treated splenocytes from mice with 1 ml erythrocyte lysis buffer (Sigma) and resuspended them in RPMI supplemented with 10% fetal bovine serum (FBS) (Hyclone, Logan, UT), beta-mercaptoethanol (Sigma, St. Louis, MO), R595 LPS (Alexis), ConA stimulated supernatant and antibiotics (penicillin, streptomycin). The ConA stimulated supernatant was made from naïve mice splenocytes cultured with 2.5 ug/ml ConA and 20 ng/ml PMA for 48 hours at 37°C in a humid atmosphere with 5% CO_2_. We then cultured spleen cells in 96 well round-bottom plates containing 1×10^7^ cells/mL irradiated syngeneic spleen cell feeders (1200 rad) from naïve mice, and 1×10^5^ cells/well from immunized mice in a total volume of 200 µl. Plates were then incubated at 37°C in a humid atmosphere with 5% CO_2_. After 6 days in culture, cells were harvested and antigen-specific memory B cell responses were measured by conventional ELISPOT method. We assessed antigen-specific OSP and total IgG ELISPOT assays on these cultured cells. Specifically, nitrocellulose bottom plates (MAHAS4510, Millipore, Bedford, MA) were coated with OSP:BSA (100 ng/well) or with goat anti-mouse IgG (Southern Biotech, Birmingham, AL) or with keyhole limpet hemocyanin (KLH; Pierce Biotechnology, Rockford, IL) (2.5 µg/mL, negative control). After we blocked the plates with RPMI supplemented with 10% FBS, we added the cultured cells to the wells and incubated the plates for 5 h at 37°C in a humid atmosphere with 5% CO_2_. We then added biotinylated anti mouse IgGγ (Southern Biotech, Birmingham, AL) antibody at 1∶1000, detected IgG antibody expressing cells using horseradish peroxidase-conjugated avidin-D (5 mg/ml, Vector Labs), and developed plates with AEC (3 amino-9-ethyl-carbozole; Sigma). We used unstimulated samples as negative controls and assessed responses to KLH. We characterized a responder as having >2 times total IgG cells with stimulation versus no stimulation and >3 anti-OSP spots.

### Neonatal challenge experiments

To assess protection afforded by immunization, we used a cholera neonatal mouse challenge assay, as previously described [10], [11], using wild-type O1 Ogawa *V. cholerae* O395. In brief, we removed three to five days old un-immunized CD-1 suckling mice (*n* = 20 mice/cohort) from dams two hours prior to inoculation. We then administered to pups a 50 µl inoculum comprised of 2.3×10^9^ CFU of *V. cholerae* O395 mixed with a 1∶250 dilution of pooled day 56 serum from mice intramuscularly immunized with the conjugate vaccine OSP:TThc with dmLT, or immunized with dmLT alone. Following oral challenge, we kept neonates separate from dams at 30°C and monitored animals every 3 hr for 36 hr, after which surviving animals were euthanized.

### Statistics and graphs

We compared data from different groups using Mann-Whitney U tests. Within each group, comparisons of data from different time points to baseline data (day 0) were carried out using Wilcoxon Signed-Rank tests. Kaplan-Meier and log rank analysis were carried out to compare survival curves in the neonatal challenge study. All reported P values were two-tailed, with a cutoff of *P*<0.05 considered a threshold for statistical significance. We performed statistical analyses using GraphPad Prism 4 (GraphPad Software, Inc., La Jolla, CA).

## Results

### Analysis of OSP:TThc

We determined progress of conjugation and average carbohydrate content/carbohydrate–protein ratio of OSP:TThc by Surface-Enhanced Laser Desorption–Ionization Mass Spectrometry (SELDI) [25]. Similar to the matrix assisted variant (MALDI) [26], this technique determines average degree of incorporation of carbohydrate onto protein, as well as molecular weight distribution in glycoconjugates. The SELDI analysis showed that the average molecular mass of the conjugate was 90,150 Da. Subtracting from that value the molecular mass of the recombinant protein TThc carrier, 52,108 Da [17] [27] the conjugate product molecular mass increased by 38,042 Da. Based on the difference between m/z values of subpeaks within the SELDI peak [14] ; also [28] the molecular mass of the polymolecular OSP–core was determined to average ∼5,900 Da, representingattachment of various lengths of OSP to core. The molecular mass of the conjugate determined by SELDI, 91,150 Da, then indicated the molar ratio of OSP–core:TThc to be ∼6.4∶1.

### OSP-specific antibody responses

**Figure 2:**
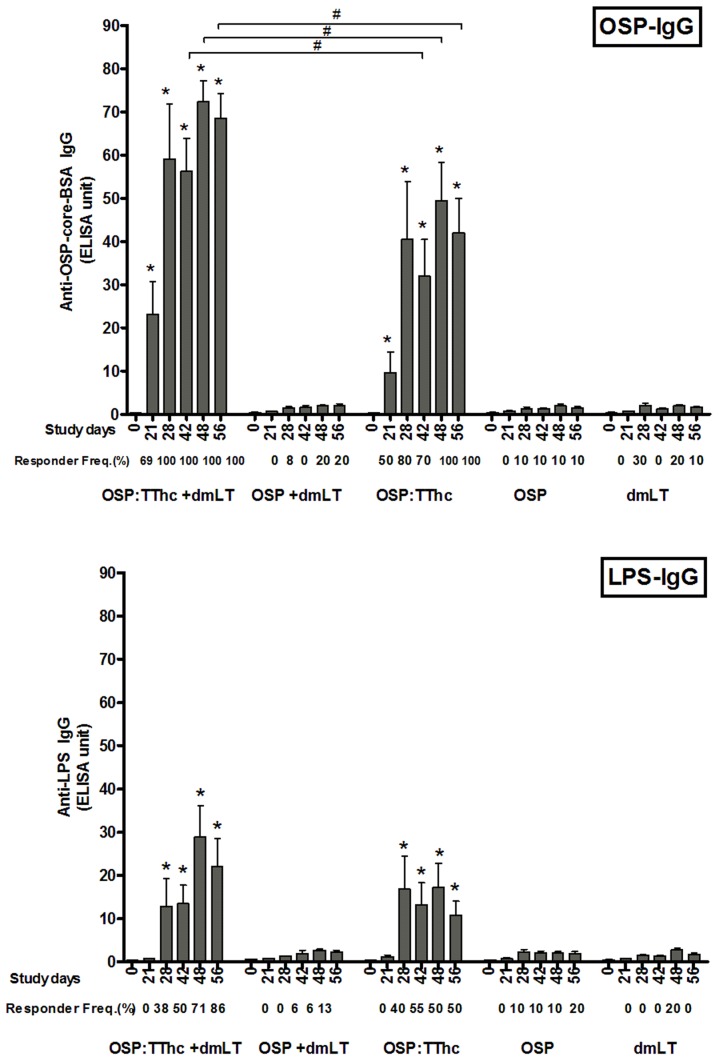
Serum anti-OSP-BSA and anti-LPS IgG antibody responses in mice intramuscularly immunized with OSP:TThc (with dmLT), OSP (with dmLT), OSP:TThc (no dmLT), OSP (no dmLT), or dmLT alone. Mean and standard error of the mean are reported for each group. An asterisk denotes a statistically significant difference (*P*<0.05) from baseline (day 0) titer. Responder frequencies are also listed. #, statistically significant difference among the compared cohorts (*P*<0.05).

**Figure 3:**
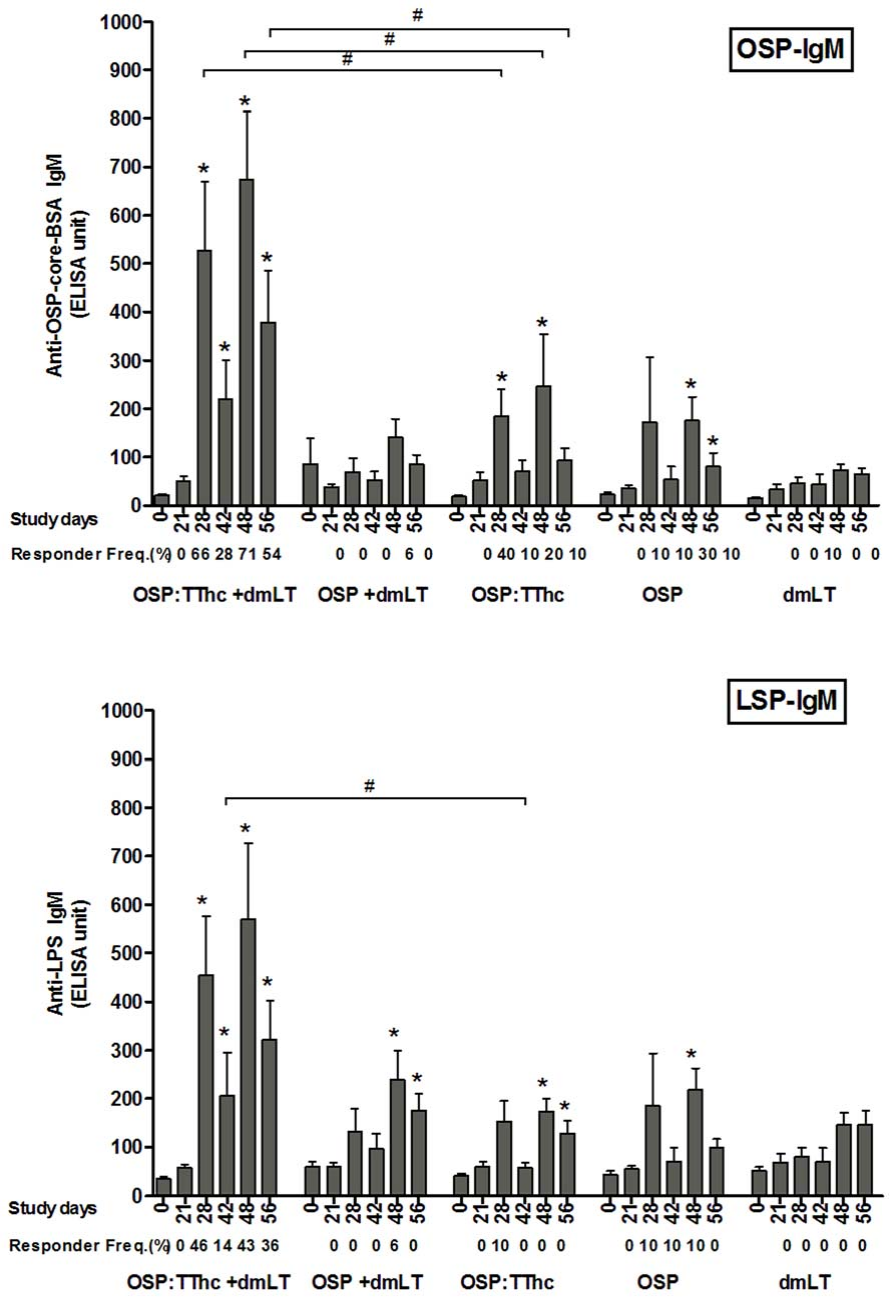
Serum anti-OSP-BSA and anti-LPS IgM antibody responses in mice intramuscularly immunized with OSP:TThc (with dmLT), OSP (with dmLT), OSP:TThc (no dmLT), OSP (no dmLT), or dmLT alone. Mean and standard error of the mean are reported for each group. An asterisk denotes a statistically significant difference (*P*<0.05) from baseline (day 0) titer. Responder frequencies are also listed. #, statistically significant difference among the compared cohorts (*P*<0.05).

Following the first injection, we detected significant anti-OSP serum IgG antibody responses in mice receiving OSP:TThc with or without adjuvant (figure 2). Higher magnitude and response rates (*P*<0.01) were observed in the cohort of animals receiving conjugate vaccine with dmLT (response rate after two doses: 100%). No anti-OSP IgG responses were detected at any time in animals receiving un-conjugated OSP only, with or without immunoadjuvant, or in animals receiving immunoadjuvant alone. Mice receiving OSP:TThc with or without immunoadjuvant and mice receiving OSP alone developed anti-OSP IgM responses (figure 3). IgM responses were only detected following a minimum of two immunizations, and response frequency and magnitude were highest in animals receiving OSP:TThc with adjuvant. No significant IgA anti-OSP antibody was detected in any group (not shown).

### LPS-specific antibody responses

Significant serum anti-LPS IgG responses developed following a second immunization in mice receiving conjugate with or without adjuvant (figure 2). Anti-LPS IgM responses were detected in all vaccine cohorts except in mice receiving immunoadjuvant alone (figure 3). No significant anti-LPS IgA responses developed in any group (not shown).

### Vibriocidal responses

**Figure 4:**
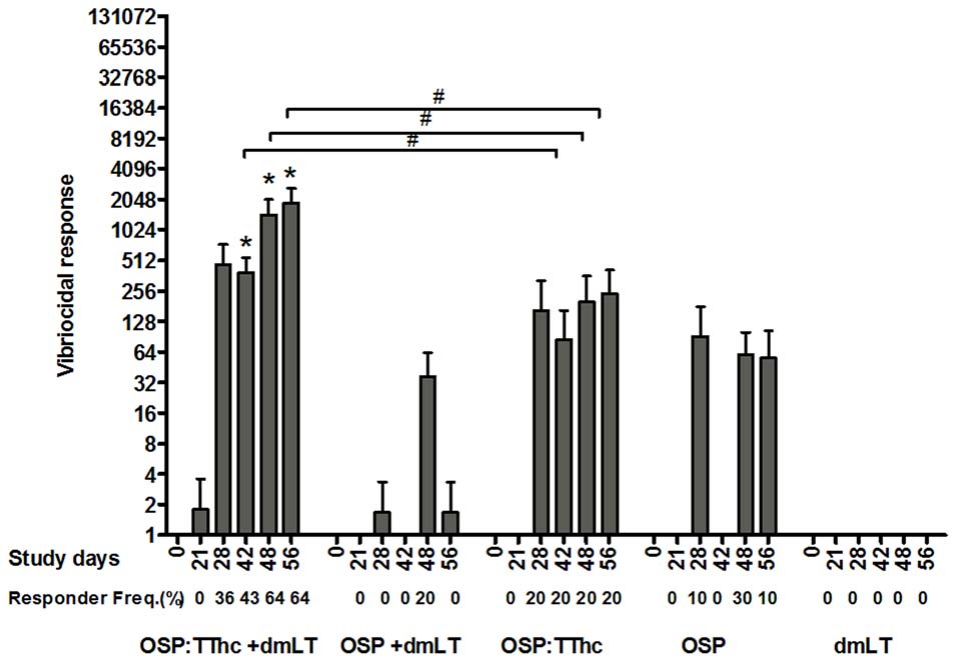
Vibriocidal responses in mice intramuscularly immunized with OSP:TThc (with dmLT), OSP (with dmLT), OSP:TThc (no dmLT), OSP (no dmLT), or dmLT alone. The columns indicate mean reciprocal end titers, and error bars represent the standard errors of the mean. An asterisk denotes a statistically significant difference (*P*<0.05) from baseline (day 0) titer. Responder frequencies are also listed. #, statistically significant difference among the vaccine cohorts (*P*<0.05).

Low-level vibriocidal responses (magnitude and response frequency) were detected in animals receiving unconjugated OSP with or without adjuvant (figure 4). Administration of the immunoadjuvant alone did not elicit any vibriocidal response in animals.

### Antigen-specific memory B cell responses

Antigen-specific IgG memory B-cell responses are shown in table 1. OSP IgG specific memory B cell responses were detected in 65% of mice immunized with conjugate vaccine and adjuvant. 18% and 22% of mice immunized with OSP in the presence of dmLT or OSP:TThc alone developed detectable OSP specific memory B cell responses, respectively. No OSP memory response was detected in mice receiving dmLT alone.

**Table 1:** OSP-BSA specific IgG memory B cell responses in mice immunized with different vaccine antigens.

Vaccine cohort	Anti-OSP	Anti-KLH
	(%)*	(%)*
**OSP:TThc+dmLT**	7/11 (65)	0/11 (0)
**OSP+dmLT**	2/11 (18)	0/11 (0)
**OSP:TThc**	2/9 (22)	0/9 (0)
**OSP**	1/9 (11)	0/9 (0)
**dmLT**	0/8 (0)	0/8 (0)

* Data are expressed as responder frequencies (see text).

### Neonatal mouse challenge assay

**Figure 5:**
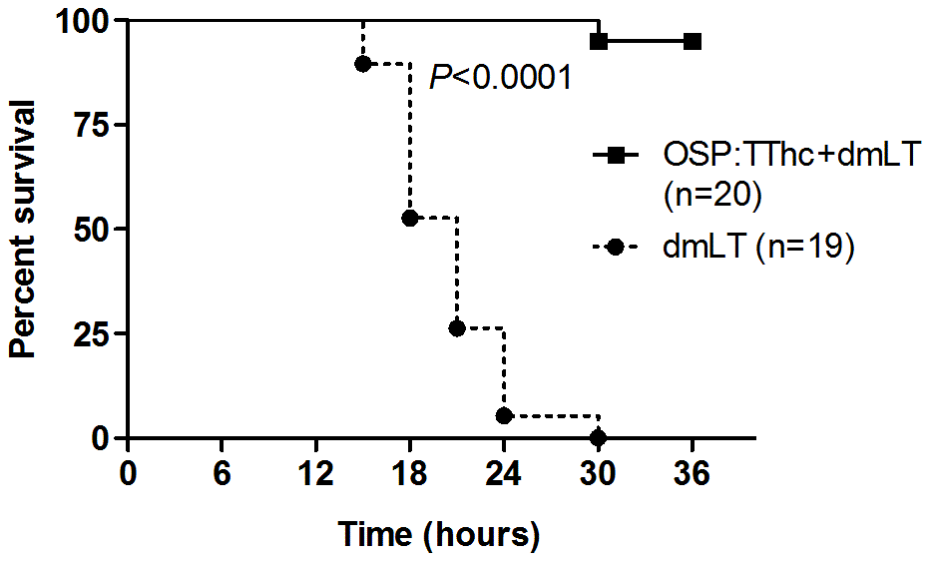
Survival likelihood of neonatal CD-1 mice following oral challenge with wild-type O1 Ogawa *V. cholerae* O395. Three- to five-day-old pups (cohort size 20) were orally gavaged with 50 µl of a preparation containing 2.3×10^9^ CFU of wild type *V. cholerae* O395 mixed with a 1∶250 dilution of pooled day 56 serum from mice intramuscularly immunized with conjugate vaccine (OSP:TThc) and immunoadjuvantative dmLT, or dmLT alone. Survival curves were compared by log rank testing.

We found a significant difference in survival between mice challenged with wild-type *V. cholerae* O1 Ogawa O395 mixed with sera collected from mice immunized with conjugate and adjuvant (95% survival at 36 hours), compared to mice challenged using sera from mice immunized with adjuvant alone (0% survival at 30 hours; 95% protection; *P*<0.0001) (figure 5).

## Discussion

In this study, we demonstrate that a cholera conjugate vaccine containing OSP recovered from *V. cholerae* is protectively immunogenic and induces anti-OSP memory B cell responses in mice. There is a growing body of evidence that anti-OSP responses may be a prime mediator of protective immunity against cholera. Protective immunity to cholera is serogroup specific. Previous infection with *V. cholerae* O1 provides no protection against O139 and vice versa. This is despite the fact that O1 and O139 express essentially identical cholera toxins (CT) and that O139 is thought to be a derivative of an O1 El Tor strain with high-level homology of most genes in O1 El Tor and O139 [29], [30]. O139 differs from O1 in its genes encoding OSP and in the presence of capsule. The capsule of O139 is comprised of a polysaccharide whose repeating unit is identical to the O139 OSP [15]. The core moieties of O139 and O1 are identical [31]. These data suggest that protection from cholera may be mediated by the serogroup OSP of LPS.

Analysis of anti-OSP responses in cholera patients and their potential role in protection has only recently been initiated [9], [32]. There is however significant evidence that anti-LPS responses correlate with protection from cholera [33], [34]. The vibriocidal response correlates with protection [35] and is largely comprised of anti-LPS IgM responses [36]. We have recently shown that the vibriocidal response can be largely adsorbed away by OSP [9]. Anti-LPS IgA responses in serum and stool have also been associated with protection against cholera among household contacts of cholera patients in Bangladesh [34]. Anti-LPS memory B cell responses similarly correlate with protection against cholera [33].

Currently, two oral killed cholera vaccines are WHO-prequalified and commercially available [37]. One contains approximately 10^11^ killed *V. cholerae* O1 classical and El Tor strain organisms (Ogawa and Inaba) and is supplemented with 1 mg of recombinant non toxic B subunit of cholera toxin (WC:rBS; Dukoral, Crucell, Sweden). The other is bivalent, containing killed classical and El Tor O1 organisms as well as an O139 strain, and it does not contain supplemental cholera toxin subunit (Shanchol, Shantabiotechnic-Sanofi, India). Following two doses, these vaccines are 40–85% effective for 6–60 months [13], [37]–[39]. The level of response and duration of protection is particularly decreased in children younger than 5 years of age, compared to older children and adults [38], [40], [41], with booster doses of Dukoral being recommended every 6 months for children under 5 years of age [42]. In comparison, wild-type cholera is associated with high level (90–100%) of protective immunity for at least 3 years in volunteer challenge studies [43] and 3–10 year protection in population-based studies [44]. The level and duration of protection afforded by previous wild-type cholera appears to be the same in young children and in older individuals [45], [46].

We have previously shown that wild-type cholera is associated with a pro-inflammatory response even in young children in Bangladesh, but that vaccination of Bangladeshi children with WC-rBS induces a T-regulatory response [47]. We have also shown that wild-type cholera induces anti-LPS memory B cell responses, even in young children [45], but that children and adult recipients of WC-rBS do not develop such responses [13], [41]. In addition, induction of memory B cell responses correlates with the magnitude of early T cell responses in older recipients of WC-rBS [47], but younger child recipients do not develop T cell responses [47]. These observations may in part explain the lower level and shorter duration of protection afforded by WC-rBS in young children compared to that induced by wild-type disease. Unfortunately, children bear a very large burden of cholera, especially in endemic areas [48], [49]. For instance, 40–80% of children in Bangladesh develop serologic evidence of previous exposure to *V. cholerae* by the age of 15 years [35], [50], and in areas of India, there is an estimated cholera incidence of 7 per 1000 for children less than 5 years of age, compared to 2.19 in older children and 0.93 in adults (>14 years age) [51]. There is thus a need for improved cholera vaccines or immunization strategies capable of inducing high-level and long-term immunity, especially in young children.

Immune responses targeting OSP may be critical in determining protective immunity from cholera. Since OSP is a T cell-independent antigen, and because young children do not develop prominent responses to polysaccharide antigens administered alone, we are particularly interested in developing a cholera conjugate vaccine. Here we show that a cholera conjugate vaccine is protectively immunogenic in mice and induces memory B cell responses against OSP. Previous prototype cholera conjugates have been developed [52] [53] [54] Our work contains a number of innovative features. The conjugation process is carried out using squaric acid chemistry, linking the glucosamine present in core oligosaccharide to carrier protein via single point attachment [14]. This takes advantage of the core oligosaccharide, effectively using it as a linker and resulting in a sun-burst display of OSP in a manner that may mimic that present on the surface of *V. cholerae*. Recent data suggest that the way LPS antigen is presented can impact subsequent immune responses [55]. The fact that the resulting conjugate in our analysis is not cross-linked and, therefore, easier to characterize, together with conjugation methodology that produces conjugates in a predictable manner [56], maximizes the likelihood that vaccine generated in this way and its immunological properties would be reproducible, which is not the case with a number of conjugate vaccines for cholera reported to date. Of note, we do not think that core oligosaccharide contributes significantly to the protective immunity that we observed since previous infection of humans with *V. cholerae* O1 does not provide protection from *V. cholerae* O139 and vice versa, despite the presence of identical core oligosaccharides. We also employed as carrier a recombinant immunogenic fragment of tetanus toxoid that could be used as carrier in other vaccines as well. Individuals at risk of cholera are often the most globally disenfranchised and impoverished and may not have received all recommended immunizations, including tetanus vaccine. In addition, we used a novel immunoadjuvant, dmLT [18]. A number of derivatives of the ADP-ribosylating LT molecule of *E. coli* have been developed and evaluated in humans [18], [57], [58]. These molecules have in common their retained immunoadjuvanticity but markedly diminished enterotoxicity [18]. We have previously shown that transcutaneously applied CT or LT can act as an immunoadjuvant [10], [11]; here we show that low-dose dmLT can also be safely administered parenterally in mice.

Our study is encouraging, but many questions remain. Would an Inaba-based vaccine result in comparable results? Would a response targeting Ogawa OSP cross protect against Inaba? Previous human suggests it may not [59] Would an Inaba-based vaccine protect against Ogawa-associated disease? Could a bi/multi-valent conjugate vaccine be developed? Our vaccine induced vibriocidal responses. Is this a reflection of the fact that a significant component of the vibriocidal response can be adsorbed with OSP [9] or are additional purification steps required? How do conjugates using purified OSP compare to glycoconjugate vaccines prepared from synthetic carbohydrates, which are also under development [10], [11], [60], [61]? Could other immunoadjuvants be used? Is it possible to induce mucosal responses, or would a parenteral cholera vaccine be sufficient when most humans at risk of cholera are also at high risk of tropical or environmental enteropathy with attendant leaking of serum antibodies into the intestinal lumen?

Despite these questions, it is notable that previously produced killed LPS-based whole cell parenteral cholera vaccines were associated with up to 80% protection against disease in humans [62]. Our data suggest that an improved parenteral cholera conjugate vaccine can be developed, one that induces immune responses, including memory B cell responses, to a normally T cell independent antigen (OPS) that is the major target of protective immunity to cholera. Furthermore, this conjugate vaccine can protect against wild-type challenge in animals. Such a conjugate vaccine could have particular utility in young children who are most at risk of cholera.
